# Cortical Representation of Lateralized Grasping in Chimpanzees (*Pan troglodytes*): A Combined MRI and PET Study

**DOI:** 10.1371/journal.pone.0013383

**Published:** 2010-10-13

**Authors:** William D. Hopkins, Jared P. Taglialatela, Jamie L. Russell, Talia M. Nir, Jennifer Schaeffer

**Affiliations:** 1 Division of Developmental and Cognitive Neuroscience, Yerkes National Primate Research Center, Atlanta, Georgia, United States of America; 2 Department of Psychology, Agnes Scott College, Decatur, Georgia, United States of America; 3 Department of Biology and Physics, Kennesaw State University, Kennesaw, Georgia, United States of America; Università di Parma, Italy

## Abstract

Functional imaging studies in humans have localized the motor-hand region to a neuroanatomical landmark call the KNOB within the precentral gyrus. It has also been reported that the KNOB is larger in the hemisphere contralateral to an individual's preferred hand, and therefore may represent the neural substrate for handedness. The KNOB has also been neuronatomically described in chimpanzees and other great apes and is similarly associated with handedness. However, whether the chimpanzee KNOB represents the hand region is unclear from the extant literature. Here, we used PET to quantify neural metabolic activity in chimpanzees when engaged in unilateral reach-and-grasping responses and found significantly lateralized activation of the KNOB region in the hemisphere contralateral to the hand used by the chimpanzees. We subsequently constructed a probabilistic map of the KNOB region in chimpanzees in order to assess the overlap in consistency in the anatomical landmarks of the KNOB with the functional maps generated from the PET analysis. We found significant overlap in the anatomical and functional voxels comprising the KNOB region, suggesting that the KNOB does correspond to the hand region in chimpanzees. Lastly, from the probabilistic maps, we compared right- and left-handed chimpanzees on lateralization in grey and white matter within the KNOB region and found that asymmetries in white matter of the KNOB region were larger in the hemisphere contralateral to the preferred hand. These results suggest that neuroanatomical asymmetries in the KNOB likely reflect changes in connectivity in primary motor cortex that are experience dependent in chimpanzees and possibly humans.

## Introduction

It has been suggested that morphological and neural adaptations for the control of the hand in primates have allowed for the evolution of complex manipulatory capacities in humans such as tool use [1], fine motor skill and precision grasping [2], [3], and speech production [4]. For these reasons, there has been significant scientific interest in understanding the evolution and development of manual motor skill in relation to the brain in humans and other primates [5].

Clinical and functional imaging studies in humans, as well as neurophysiological studies in nonhuman primates have identified several cortical regions involved in reaching and grasping movements [6], [7]. Notably, areas within the premotor and primary motor cortex as well as the superior parietal lobe have been shown to be active when subjects are required to grasp objects [8], [9], [10]. In humans, an area within the dorsal portion of the precentral gyrus has been identified as an important neurobiological correlate of handedness [11], [12], [13]. Specifically, structural magnetic resonance imaging (MRI) studies have identified a morphological landmark in the precentral gyrus, referred to as the KNOB, which is larger in the hemisphere contralateral to the subjects' preferred hand [14]. Similarly, unilateral activation of the KNOB and adjacent regions are observed when subjects perform grasping responses with the left or right hand or when these appendages are stimulated [15], [16]. The structural and functional link between handedness and variation in lateralization in the KNOB has led some to suggest that this brain region may serve as the neurobiological substrate for handedness in humans and possibly other primates [14], [17], [18].

Recently, the KNOB has been anatomically identified in all four species of great apes, but is absent in lesser apes, as well as in Old and New World monkeys. This observation suggests that there has been evolutionary changes in cortical organization of the precentral gyrus that may be a consequence of selection for increasing motor control of the hand in great apes compared to other primates [17]. The presence of the KNOB in great apes but not other nonhuman primates is consistent with existing behavioral data showing species difference in object manipulation skills [19], tool using variation and diversity [20], and more pronounced preshaping and prehension abilities compared to monkeys [21], [22]. In addition, individual variation in the lateralization of the KNOB of chimpanzees at both the anatomical and cellular level of analysis is associated with handedness in chimpanzees with right-handed individuals having a larger left KNOB compared to left-handed individuals [23], [24], [25]. These results suggest that the KNOB may similarly be the anatomical substrate for handedness, not just in humans, but also in chimpanzees (and possibly other primates) [26]; however, there are several theoretical and methodological issues that limit the interpretation of these existing findings.

First, although handedness is correlated with asymmetries in the KNOB of chimpanzees, whether or not this region of the precentral gyrus is directly involved in the motor control of the hand is unclear and cannot be determined from morphological studies alone. Previous studies have shown that electrical stimulation of certain regions of the precentral gyrus elicit finger movements in chimpanzees [27] and large scale lesions of this region results in transient disruption of motor and prehensile functions [28]. However, these early lesion and stimulation studies were terminal and included the use of only a few subjects thus considerably limiting the conclusions that can be drawn from these data. With the advent of modern imaging technologies, scientists now have an opportunity to directly assess neurofunctional correlates of motor functions using non-invasive *in vivo* techniques in behaving animals. One goal of the current study was to evaluate regional cortical activation using positron emission tomography (PET) in chimpanzees performing a reach-and-grasp task. Recent studies have shown that PET can be used successfully in chimpanzees to image neural activity associated with cognitive and communicative functions including gestural communication [29], auditory perception of species-specific sounds [30], and discrimination of facial expressions [31]. Our aim in using PET in this study was to determine if the KNOB is significantly activated when chimpanzees produce prehensile reaching-and-grasping actions. For this purpose, we required that the chimpanzees produce unilateral reach-and-grasp responses during the PET uptake period. Our specific hypothesis was that unilateral activation of the motor-hand area would be evident in the hemisphere contralateral to the hand used for reaching and grasping by the chimpanzees.

**Figure 1:**
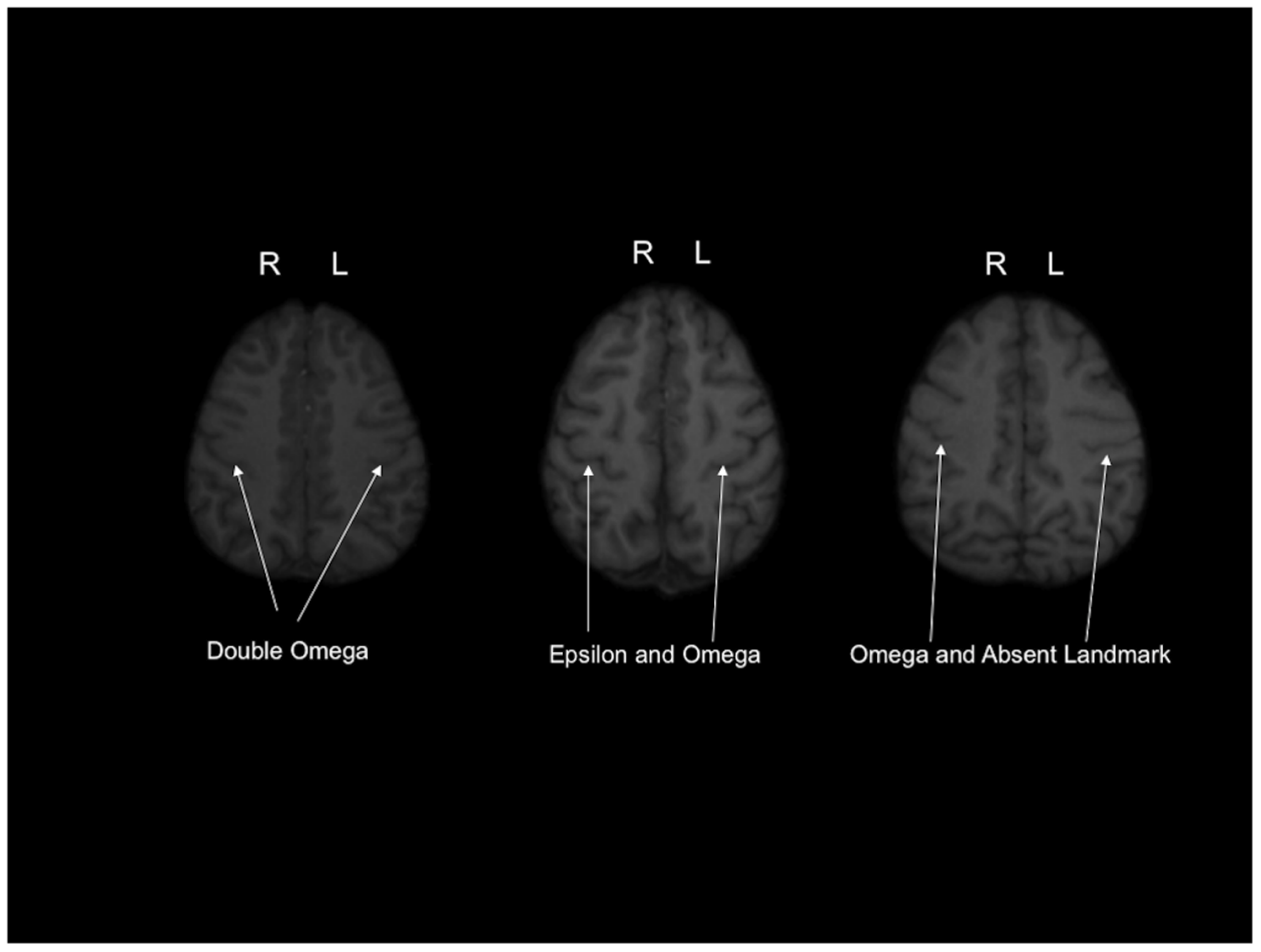
Axial views of MRI scans of chimpanzee brains showing different variants of the sulci used to define the motor hand area as described by Caulo et al. [**32**] in human subjects. The “omega” shaped KNOB is the most common variant.

In chimpanzees, as with humans [32], there is considerable variability in the sulci used to define the KNOB (see Figure 1). The KNOB has been described as an inverted omega or epsilon shaped sulcal protrusion that emerges as a consequence of the gyrus caudally displacing the central sulcus [15], [33] (see Figure 1). The variability in the presence and/or size of the KNOB across subjects makes it difficult to quantify this region anatomically, and thereby limits the extent that it can be used as a potentially reliably indicator of handedness. To address the issue of sulcal variability, a second goal of this study was to create a probabilistic map of the motor-hand region in chimpanzees as a means of characterizing individual variability (and consistency) in this region. We subsequently combined the probabilistic mapping data with the functional imaging data derived from the PET experiment described in the previous paragraph to evaluate whether the functional areas involved in reach-and-grasping movements overlap with the morphological probabilistic map of the KNOB region. The aim of this analysis was to directly assess the relation between the structure and function of the KNOB region in the chimpanzee brain.

Lastly, because our goal was to attempt to link lateralized reach-and-grasping movements using PET with anatomical variation in the KNOB region, we also examined whether performance differences in grasping skill between the left and right hands were associated with variation in lateralization of the motor-hand area. Rather than focus on the entire volume of the KNOB region, in this study we examined whether the variation in hand skill were linked to lateralization in grey and white matter within the KNOB. When considering grey and white matter separately, anatomical studies in humans suggest that asymmetries in white matter within the KNOB region are associated with handedness. For example, Buchel et al. (2004) used diffusion tensor imaging to assess white matter asymmetries in human MRI scans and found significantly leftward asymmetries in white matter underneath the KNOB region in sample of right-handed individuals. In another study, Lu et al. [34] examined cortical thickness in the KNOB region as well as the inferior formal gyrus in developing children in relation to the motor skill and phonological development. With respect to the KNOB, Lu et al. found that increasing development of handedness in motor skill was associated with decreasing cortical thickness in the hemisphere opposite to the preferred hand. In this case, the reduced cortical thickness may reflect increased white matter development in the KNOB region in relation to increasing motor skill development.

Previously, we have found that captive chimpanzees make significantly fewer errors with the right compared to left hand when grasping small food items [35], [36]. If our hypothesis is correct and the KNOB region is involved in prehensile grasping, then potential anatomical differences in the size of the motor-hand region should be associated with performance differences between the hands, as has been reported in human subjects [37], [38]. This hypothesis was tested by comparing the lateralization of the KNOB in chimpanzees who performed better with the left and right hands on the grasping tasks. Although neuroanatomical asymmetries in hand preference have been examined in chimpanzees and monkeys [18], [24], [26], [39], [40], [41], [42], [43], [44], [45], [46], this is the first study that has explicitly examined the neuroanatomical correlates of functional asymmetries in manual performance asymmetries in nonhuman primates. We emphasize this aspect of the study because, although the assumption is that hand preferences are a consequence of inherent specializations of the left or right hemisphere, hand preferences do *not* strongly correlate with performance asymmetries in humans subjects [47], [48], [49], [50], [51]. Thus, to some extent, hand preference and performance may assess different dimensions of hemispheric specialization.

## Results

### PET Analyses

#### Descriptive statistics

The frequency of the left and right hand grasping responses during the uptake period by each chimpanzee are shown in Table 1. As can be seen, the chimpanzees produced a substantial number of reach-and-grasp responses and the majority of them were unilaterally produced by the assigned hand. Thus, the execution of unimanual reaching was accomplished during the uptake period.

**Table 1:** Frequency of left and right hand grasping responses for each subject during FDG uptake period.

Subject	Left Hand Responses	Right Hand Responses
Dara	0	3-3
Rowena	453	6
Jolson	460	7
Edwina	380	7

#### Whole brain analysis

**Figure 2:**
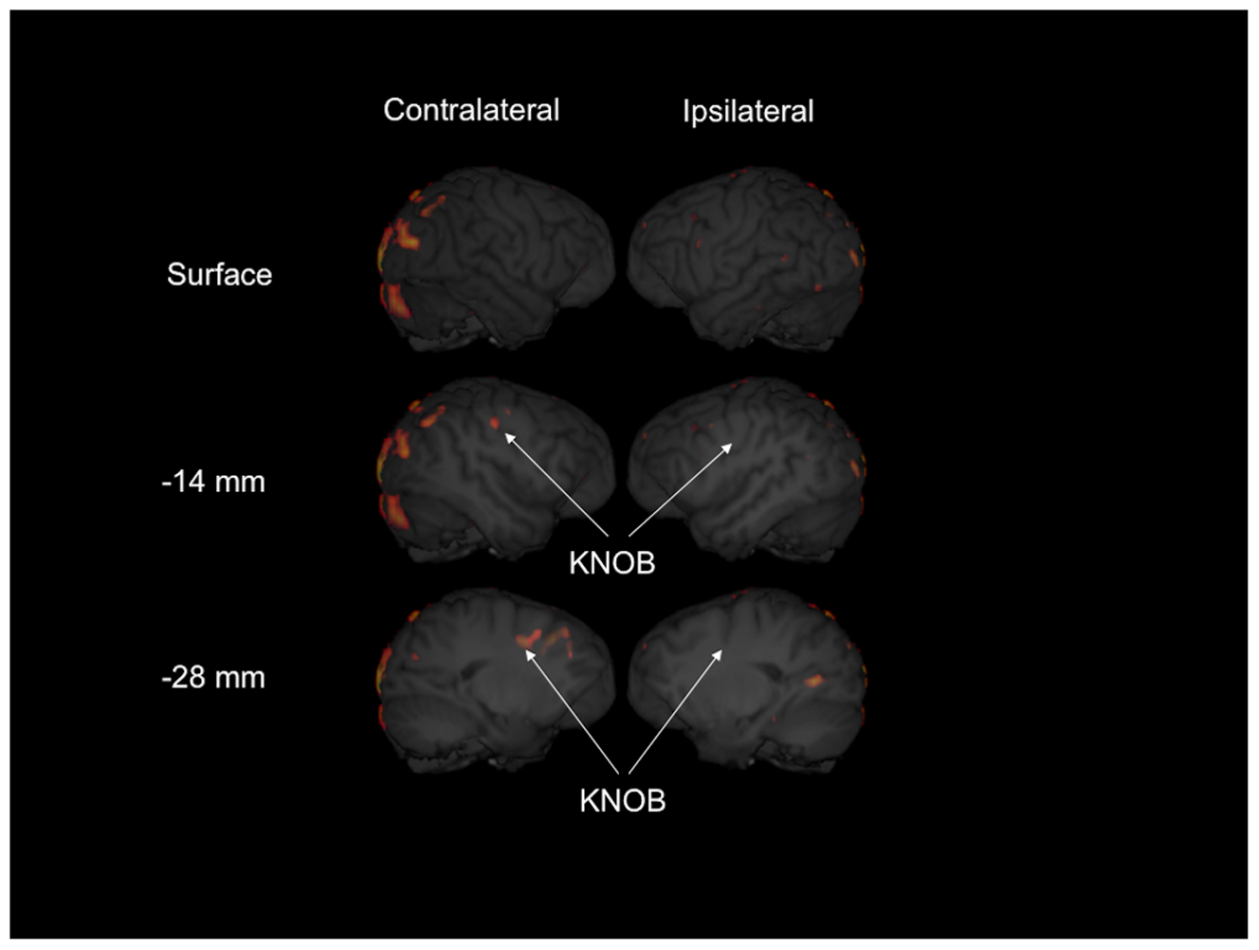
3D reconstructions of chimpanzee MRI scan with PET activation *t*-map distribution for the contralateral and ipsilateral hemispheres projected onto the surface and at two distances (+/− 16 mm, +/− 28 mm) from the midline in the sagittal plane of view. “x”, “y,” and “z” indicate the orthogonal planes (sagittal, coronal, and axial respectively) and arrow directions refer to ascending slices as depicted in the left panel of the figure.

A 3-D rendering with the significant areas of activation identified from the whole brain analysis of the PET data is shown in Figure 2. In total, 10 significant clusters were identified, 5 in the hemisphere contralateral to the grasping hand, 1 in the ipsilateral hemisphere, and 4 were found bilaterally. The x, y, z coordinates and associated *t*-scores are shown in Table 2. X-coordinate values indicate the distance from the mid-sagittal plane (in mm). Negative values indicate the contralateral hemisphere, positive values are localized to the ipsilateral hemisphere. Y- and Z- coordinate values refer to the distance (in mm) from the frontal pole and most dorsal portion of the brain, respectively (see Figure 2 for reference image). There was also one significant cluster found in the lateral portion of the cerebellum in the contralateral hemisphere. Of particular note was the significant cluster found in the region corresponding to the KNOB in the hemisphere contralateral to the hand used for grasping (see Figure 2). In addition, significant clusters in the contralateral hemisphere were found for the medial and ventral premotor areas, dorsal primary motor cortex, and the superior frontal gyrus (see Figure 2 and Table 2).

**Table 2:** Significant regions of PET activation in the contralateral and ipsilateral hemispheres to the hand used for reach-and grasp responses.

Region	t	X	Y	Z
*Contralateral*				
Motor-Hand/KNOB	5.48	−30	56	22
Dorsal Precentral Gyrus	5.73	−.28	50	16
Dorsal Middle Frontal Gyrus	5.70	−14	22	26
Ventral Premotor Cortex	5.87	−20	42	24
Occipital Lobe	8.36	−12	106	42
Supramarginal Gyrus	6.28	−30	84	18
Precuneus	5.06	−16	92	32
Superior Frontal Gyrus	5.70	−10	36	16
Superior Parietal Cortex	6.93	−12	92	14
*Ipsilatera*l				
Superior Frontal Gyrus	5.67	4	42	18
Superior Parietal Cortex	5.71	20	92	22
Occipital Lobe	7.02	20	104	42
Ventral Premotor Cortex	5.37	32	36	42
Lingual Gyrus	6.36	18	88	42

### Neuroanatomical Probabilistic Map of KNOB region

#### Descriptive statistics

The volumes of the KNOB object maps at the 30%, 50%, and 70% thresholds for the left and right hemispheres are shown in Table 3. In general, although there was a slight leftward bias across thresholds, these differences were not particularly large. For the sake of completeness, the percentage of GM and WM for the left and right KNOB regions is shown in Table 3. To reduce the number of analyses performed on the overall data, we initially performed a correlation between the percentage of GM and WM to assess to what extent these measurements were redundant. Because we quantified an entire gyrus, we suspected that the proportion of GM would be inversely associated with the amount of WM and this was borne out in the subsequent analyses. Correlations between GM and WM were significant and negative at 30% (*r* = −.971, *p*<.001), 50% (*r*  =  −.977, *p*<.001) and 70% (*r*  = −.985, *p*<.001) threshold criteria. For this reason, we subsequently focused the remaining analyses on the WM data.

**Table 3:** Probabilistic volumes at three threshold levels and percentage of grey and white matter.

			Threshold Level			
	Right 30%	Left 30%	Right 50%	Left 50%	Right 70%	Left 70%
Probabilistic volume	522	631	174	279	49	179
Females GM	39.29	37.71	42.31	38.71	46.54	49.44
s.e.	(14.13)	(13.74)	(20.57)	(19.61)	(27.75)	(22.90)
Males GM	39.27	38.36	40.59	35.82	46.14	40.50
s.e.	(16.95)	(13.19)	(23.14)	(17.71)	(28.59)	(20.35)
Females WM	60.96	62.63	57.81	62.31	53.42	61.56
s.e.	(15.02)	(14.24)	(21.68)	(19.89)	(29.04)	(23.18)
Males WM	59.91	60.82	58.45	59.68	53.14	58.77
s.e.	(17.65)	(13.79)	(24.03)	(18.00)	(27.71)	(20.73)

Values in parentheses are standard deviations. Probabilistic volumes are in mm^3^.

### Performance Asymmetries

We initially examined whether this sample fo chimpanzees showed population-level biases in hand performance in the grasping task using a one-sample *t*-tests. A significant population-level right hand bias was found *t*(69) = 3.07, *p*<.001 (Mean HI  = −.137). Independent samples *t*-tests failed to significant sex differences in either the HI measure or in the percentage of grasping error made by the chimpanzees. Based on the sign of the HI values, 40 chimpanzees were judged to perform better with the right hand compared to 28 with their left hand.

### Sex and Handedness Effects

**Figure 3:**
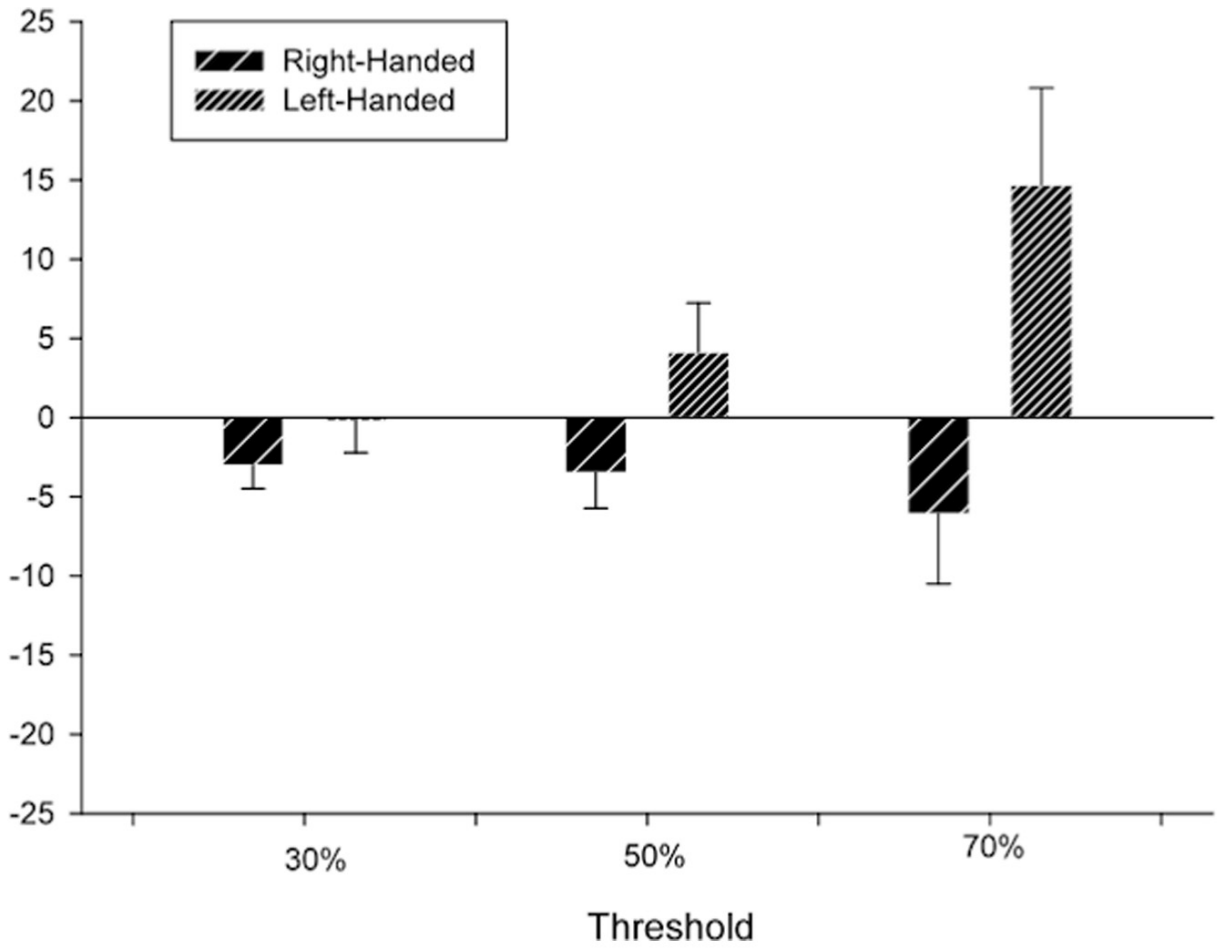
Mean AQ (+/− s.e.) in WM for chimpanzees who perform significantly better on grasping with the right (right-handed) and left hands (left-handed) at the three different probabilistic thresholds. Positive values indicate greater rightward asymmetries while negative values greater leftward biases.

For this analysis, we compared the asymmetry quotients (AQ) in WM as a function of threshold, sex and handedness. Sex and handedness were the between group factors while threshold (30%, 50%, 70%) was the repeated measures. The AQ were computed following the formula [AQ  =  (R – L)/((R + L) *5)] where L and R reflect the percentage white matter for the left and right hemispheres. Thus, positive AQ values indicated a rightward bias while negative values indicated a leftward bias. A significant two-way interaction was found between handedness and threshold *F*(2, 132)  = 4.10 *p*<.02. Shown in Figure 3 are the mean AQ values as a function of hand performance. Overall, chimpanzees that make fewer errors in grasping with the right (referred to as right-handed) compared to left hand (referred to as left-handed) show greater leftward asymmetries in WM within the KNOB, particularly at the 70% threshold level.

### Combining the PET and Probabilistic Map of the KNOB

We next considered the relation between the functional PET activation patterns within the motor-hand area and the proportion of grey matter for the different probabilistic maps derived from the anatomical data using essentially a voxel-of-interest approach to the analysis. Recall from the PET analysis that there was a significant cluster of activation found in the region corresponding to the KNOB (see Table 2) as well as in regions adjacent to the KNOB region, such as the ventral premotor cortex. We isolated and created tracings of these two clusters. We then flipped the resulting map in the left-right plane, and fused it with the normally oriented maps to create bilateral tracings corresponding to the KNOB and ventral premotor cortex PET activation clusters.

For each subject, we then placed these bilateral tracings (corresponding to the KNOB and ventral premotor clusters) on each subject's segmented GM volumes. The proportion of GM found within these tracings was then computed, as was done with the anatomical probabilistic maps. The percentages of GM for the KNOB and ventral premotor cortex clusters were then correlated with the GM percentages derived from the anatomical probabilistic maps of the KNOB. Percentage of GM at the 30% (*r* = .385, *p*<.001), 50% (*r* = .322, *p*<.008) and 70% (*r* = .257, *p*<.04) thresholds were significantly positively correlated with the percentage of GM found in the KNOB cluster identified from the PET analysis. In contrast, the percentage of GM at the 30% (*r* = −.096, *p*>.05), 50% (*r* = −.110, *p*>.05) and 70% (*r* = −.110, *p*>.05) thresholds were not significantly correlated with the percentage of GM found in ventral premotor region cluster. Thus, variation in the proportion of grey matter found in the PET cluster corresponding to the KNOB region were more strongly correlated with the proportion of grey matter of the KNOB region estimated from the probabilistic map.

## Discussion

Several important findings emerged from this study. First, as we hypothesized, PET analysis of unilateral reach and grasp responses by chimpanzees revealed significant contralateral activation in several key motor areas including the motor-hand area of the precentral gyrus and associated premotor regions. Second, based on a probabilistic map of the KNOB, the proportion of grey matter corresponding to this region significantly positively correlated with the proportion of grey matter found when using a voxel-of-interest analysis derived from the PET data. Third, based on a anatomical probabilistic map of the KNOB region, chimpanzees that make fewer grasping errors with their right compared to their left hand have larger leftward asymmetries in white matter. This is particularly evident at the highest threshold of the probabilistic map, where the greatest overlap in voxels corresponding to the motor-hand regions are found across subjects.

With respect to the PET results, the findings reported here, particularly with regard to the motor-hand area, are consistent with previous results in human subjects showing functional activation in the KNOB region contralateral to the grasping hand [8], [11], [15], [52], [53] as well as single cell recording studies in monkeys [54], [55]. Thus, within the precentral gyrus, grasping actions can be localized to the motor-hand area in the chimpanzee brain and this likely represents the arm/hand region. With that stated, it is not possible to precisely isolate the cortical representation of hand movements given that the reach and grasp actions required the chimpanzees to use their entire forearm and digits when picking up the objects during the uptake period.

A second finding from this study was the significant association found between the grey matter volumes when calculated from the VOI derived from the PET data in relation to volumes derived from probabilistic maps based on the neuroanatomy of the KNOB. Specifically, when we created tracings of the KNOB region based on the functional regions involved in reaching-and-grasping responses and correlated the percentage of GM within these regions with the GM found within the anatomically defined KNOB (based on probabilistic maps), significant positive associations were found. Moreover, the spatial location of the motor-hand region derived from PET corresponded strongly with the anatomical region, even at the highest threshold level. These two independent sets of observations strongly suggest that functional data correspond well to variation in the anatomy of the motor-hand area.

Third, the results presented here indicate that variation in white matter volumes comprising the KNOB region are associated with differences in grasping performance between the right and left hands. Specifically, chimpanzees that perform better with their right hand have a higher proportion of white matter in the left hemisphere while those that perform better with their left hand have larger white matter volumes in the right hemisphere. This finding indicates that performance differences between the hands in grasping are potentially due to increased connectivity into the KNOB region, likely from sub-cortical and brain stem structures known to be involved in precision movements, such as the cortico-spinal tract or potentially other cortical regions [56]. Difference in white matter asymmetries within the KNOB region have similarly been reported in humans [57]. The patterns of results reported here are consistent with these observations and provide further support that the neural mechanisms underlying handedness are comparable in humans and chimpanzees.

Several recent studies have employed PET in chimpanzees to assess the neural correlates of face perception, auditory perception of species-specific sounds and communicative signaling [29], [31] and the results reported here provide further evidence that *in vivo* functional imaging can be conducted in apes and produce reliable results. We would further add that the effectiveness of PET in imaging behavior in chimpanzees is particularly strong in this report because we intentionally tried to elicit unimanual motor activation in the apes and this was clearly evident in our results. Thus, in some ways, the results reported here validate the use of PET for establishing structure-function relationships in chimpanzees and likely other nonhuman primates.

### Limitations and Caveats

It could be argued that the absence of a comparison scan in our subjects, which could have been subtracted from the grasping scan as a means of removing general neuronal metabolic activity, limits the interpretation of the PET results in our study. We would argue against this position because the ipsilateral hemisphere was effectively the “comparison” in this study as we intentionally tried to limit the activation of this hemisphere. With that said, in no way are we suggesting that ipsilateral brain regions are not involved in contralateral motor movements [58], [59], but this direct issue was beyond the scope and aims of this experiment. Furthermore, previous “resting state” results reported in chimpanzees, macaques and baboons [60], [61] have failed to report any evidence of asymmetrical activation in the precentral gyrus, specifically within the region corresponding to the motor-hand area. Thus, it is unlikely that the main PET results reported here can be attributed to individual variation in resting state rather than activation associated with the reach-and-grasping actions made by the chimpanzees.

### Concluding Remarks

In conclusion, reach-and-grasp responses in chimpanzees are associated with contralateral activation of the primary motor and premotor cortices, particularly in regions corresponding to the motor-hand area. PET activation of the motor-hand area correlated with the anatomical location of the motor-hand area in the chimpanzee based on a probabilistic map of this region. Finally, performance asymmetries in grasping in chimpanzees were associated with anatomical lateralization in the motor-hand area. Collectively the results reinforce the view that individual differences in hand preference and performance are linked to variation in the motor-hand area, not just in humans but also in chimpanzees. Collectively, the results suggest functional, structural and behavioral homology of the motor-hand regions in humans and chimpanzees.

During primate evolution, the brain has become increasingly large relative to body size and consequently resulted in increasing gyrification or cortical folding of the cortex [62], [63]. The KNOB represents one of these cortical folds and, in light of the observation that it can only be visualized in great apes and human brains, suggests that increasing selection for complex manual actions resulted in, in essence, a neuroanatomical foot print of that evolutionary process. Furthermore, the fact that the KNOB appears to be experience dependent [37] and may reflect the development of connections between the primary motor cortex and sub-cortical structures known to be involved in prehension, suggests that it also represents a potentially important marker of brain and behavioral development. Additional studies that focus on the role that different motor experiences have on the development of the KNOB [64], [65] would be instrumental in understanding the foundations of handedness in human and nonhuman primates.

## Methods

### Subjects

All subjects in the PET and MRI studies were captive born chimpanzees from the Yerkes National Primate Research Center (YNPRC). For the PET study, the subjects were four adolescent chimpanzees including one male (Jolson) and 3 females (Rowena, Edwina, Dara). The subjects ranged in age from 14 to 18 years of age. For the MRI studies, the sample consisted of 70 captive chimpanzees including 22 males and 48 females. Within this cohort, the subjects ranged in age from 6 to 51 years (Mean  = 21.52, s.d.  = 11. 59). All of the research conducted in this study were conducted according to the American Psychological Association guidelines for the ethical treatment of animals and were approved by the Institutional Animal care and Use Committee of Emory University. The YNPRC is fully accredited by the American Association for Accreditation of Laboratory Animal Care.

### Procedure (Positron Emission Tomography)

Subjects were scanned using ^18^F-fluorodeoxyglucose (^18^F-FDG) at a dose of 20 mCi. FDG was selected as the ligand because of its relatively long uptake period (∼80 minutes) and long half-life (approximately 110 minutes). Thus, just as we and other investigators have previously done, we capitalized on these features of ^18^F-FDG because they allowed for prolonged behavioral testing during the uptake period and a relatively long time frame to capture neural activity trapped in the cells between the termination of uptake and the interval of time needed to transport and scan the chimpanzees. Previous studies have used identical procedures to scan chimpanzees and have revealed significant and consistent patterns of PET activation (Taglialatela et al., 2008; Taglialatela et al., 2009). At the onset of behavioral testing, subjects were orally administered 20 mCi of ^18^F-fluorodeoxyglucose (FDG) that was diluted in approximately 100 ml of a sugar free flavored drink mixture. The subjects then participated in the behavioral task for 40 minutes (see below for description). Following the uptake period, chimpanzees were asked to voluntarily present for an intramuscular injection of Telazol (4 mg/kg). Training for voluntary intramuscular administration of the immobilization agent was done prior to testing, so that the chimpanzees were highly familiar with the procedure. Once anesthetized, the chimpanzees were transported to the PET imaging facility (total time from initial anesthesia induction to scan onset was ∼45 minutes). For the duration of the PET scan, chimpanzees remained anesthetized with propofol administered intravenously and diluted in lactated ringers at a dose of ∼10 mg/kg/hr. After completing PET scanning procedure, the subjects were returned to the YNPRC and temporarily housed in a single cage for approximately 18 h to allow the effects of the anesthesia to wear off and radioactivity to decay. Subjects were then returned to their home cages with their social group.

### PET Image Acquisition

The PET images were acquired on a High Resolution Research Tomograph (CPS HRRT; CTI/Siemens, Inc.) approximately 1 hour and 45 minutes following ingestion of the ^18^F-FDG. Scan procedures were identical for all subjects. Chimpanzees fasted for approximately 5 hours prior to ^18^F-FDG administration, and were rewarded with only minimal amounts of frozen sugar free flavored drink cubes during the uptake period. Subjects were placed in the supine position inside the scanner. Six minute transmission scans were followed by 20 minute emission scans. Scan parameters were identical for all subjects: Axial FOV  = 24 cm; Transverse FOV  = 31.2 cm; Slice thickness  = 1.21875 mm. Transaxial Spatial Resolution FWHM is 2.4 mm at the center and 2.8 mm 10 cm from the center. Following scanning, a post reconstruction 2 mm smooth was applied to the images.

### Behavioral Task (PET)

During the FDG uptake period, subjects were required to unilaterally grasp small rocks placed in their enclosure. Prior to testing, the subjects were separated from their social groups, but remained in their home enclosure. Three subjects (Rowena, Jolson, Edwina) used their left hand to grasp, whereas the remaining ape (Dara) used her right hand. During each test block, the experimenter would place 20 rocks in the enclosure and the subjects were required to grasp each rock, one by one, and hand them back to the experimenter. After each test block, a one-minute inter-block-interval (IBI) occurred during which the subjects remained seated quietly. After the one-minute IBI, another 20-rock test block was administered. The total number of test blocks varied across subjects but for all subjects, unilateral grasping was nearly uniformly accomplished across the entire uptake period (see Table 1). In order to maintain subject motivation during the 40-minute uptake period, the chimpanzees were given a small, frozen sugar free kool-aid ice-cube and squirt of juice at the end of each block. Subjects were also verbally praised during testing by the experimenter when they retrieved all the rocks. During the IBI, the experimenter remained seated in front of the subject but interacted as little as possible with the chimpanzee.

### PET Image Processing

The individual PET images were spatially aligned to their respective MRI images using 3D voxel registration with a linear transformation (Analyze 8.1, Mayo Clinic). Once aligned, each subject's MRI was used to outline the brain on the PET image in each and every slice in the axial plane. An average PET activation was then calculated based on the registered activity within these slices. Once the mean activation for the whole brain had been computed, each voxel within that entire volume was divided by the mean activation in order to obtain a standardized PET image. Next, images were smoothed with a low pass filter with an isotropic 6 mm kernel. To assess asymmetries in PET activation, the individually registered PET volumes were flipped on the left-right axis. The flipped PET volumes were registered back to the chimpanzee template and the flipped PET image was subtracted from the normal PET scan to create a difference volume. For the chimpanzee that used it right hand to grasp the object, the difference volume was reversed so that its left hemisphere difference values were aligned with the right hemisphere values for the remaining three subjects. This was done so that the hemisphere could be compared along the contralateral and ipsilateral dimension within the same sets of images. The four difference volumes were then spatially registered to one another, and a single average PET volume calculated. Whole brain analysis was conducted for the 4 subjects collectively using Analyze 8.1 (Mayo Clinic, Mayo Foundation, Rochester, Minnesota, USA). Significant areas of activation were identified by calculating a single t-map volume and using a threshold value of *t*  = 4.54 and a minimum cluster size corresponding to 72 mm^3^.

### MRI Image Collection and Procedure

Scans were obtained at the time the chimpanzees were being surveyed for their annual physical examinations. For all scans, subjects were first immobilized by ketamine (10 mg/kg) or telazol (3–5 mg/kg) and subsequently anaesthetized with propofol (40–60 mg/(kg/h)) following standard procedures at the YNPRC. Subjects were then transported to the MRI facility. The subjects remained anaesthetized for the duration of the scans as well as the time needed to transport them between their home cage and the imaging facility (total time ∼1.5 h). Subjects were placed in the scanner chamber in a supine position with their head fitted inside the human-head coil. Scan duration ranged between 40 and 60 min as a function of brain size.

The chimpanzees were scanned using a 3.0 T scanner (Siemens Trio, Siemens Medical Solutions USA, Inc., Malvern, Pennsylvania, USA) at the YNPRC. T1-weighted images were collected using a three-dimensional gradient echo sequence (pulse repetition = 2300 ms, echo time = 4.4 ms, number of signals averaged = 3, matrix size  = 320×320, with .6×.6×.6 resolution). After completing MRI procedures, the subjects were returned to the YNPRC and temporarily housed in a single cage for 6–12 h to allow the effects of the anesthesia to wear off, after which they were returned to their home cage. The archived MRI data were transferred to a PC running Analyze 8.1 software for post-image processing.

### Brain Region of Interest and Image Segmentation

Individuals tracing the ROIs were blind to the sex and handedness of the chimpanzees. Prior to the measurement of the KNOB, inter-rater reliability was established between the two observers. To assess inter-rater reliability, two individuals measured the KNOB for 10 individual chimpanzees following the criteria and landmarks outlined for this study (see below). The volume measures of the left and right hemispheres were correlated within each individual chimpanzee between the two raters. For the KNOB region, inter-rater reliabilities for the left (r = .96, df = 8, p<.01) and right hemispheres (r = .85, df = 8, p<.01) were positive and significant.

Prior to tracing the KNOB on each individual brain, the skulls were removed from the raw MRI scans and the brains were re-aligned in the axial planes and subsequently virtually cut into consecutive 1 mm slices [66]. Each subject's brain volume was then co-registered to a template of a chimpanzee brain using three-dimensional voxel registration with a linear transformation (Analyze 8.1). The chimpanzee brain template was created using a two-tiered procedure [61]. Initially, the MRI scans of 8 chimpanzees (subjects included in this study) were placed in stereotaxic orientation using AFNI software and then averaged together into a single image. Next, each individual MRI scan was spatially normalized to this template using an affine transformation. Subsequently, all the spatially normalized MRI scans were averaged to create the template.

**Figure 4:**
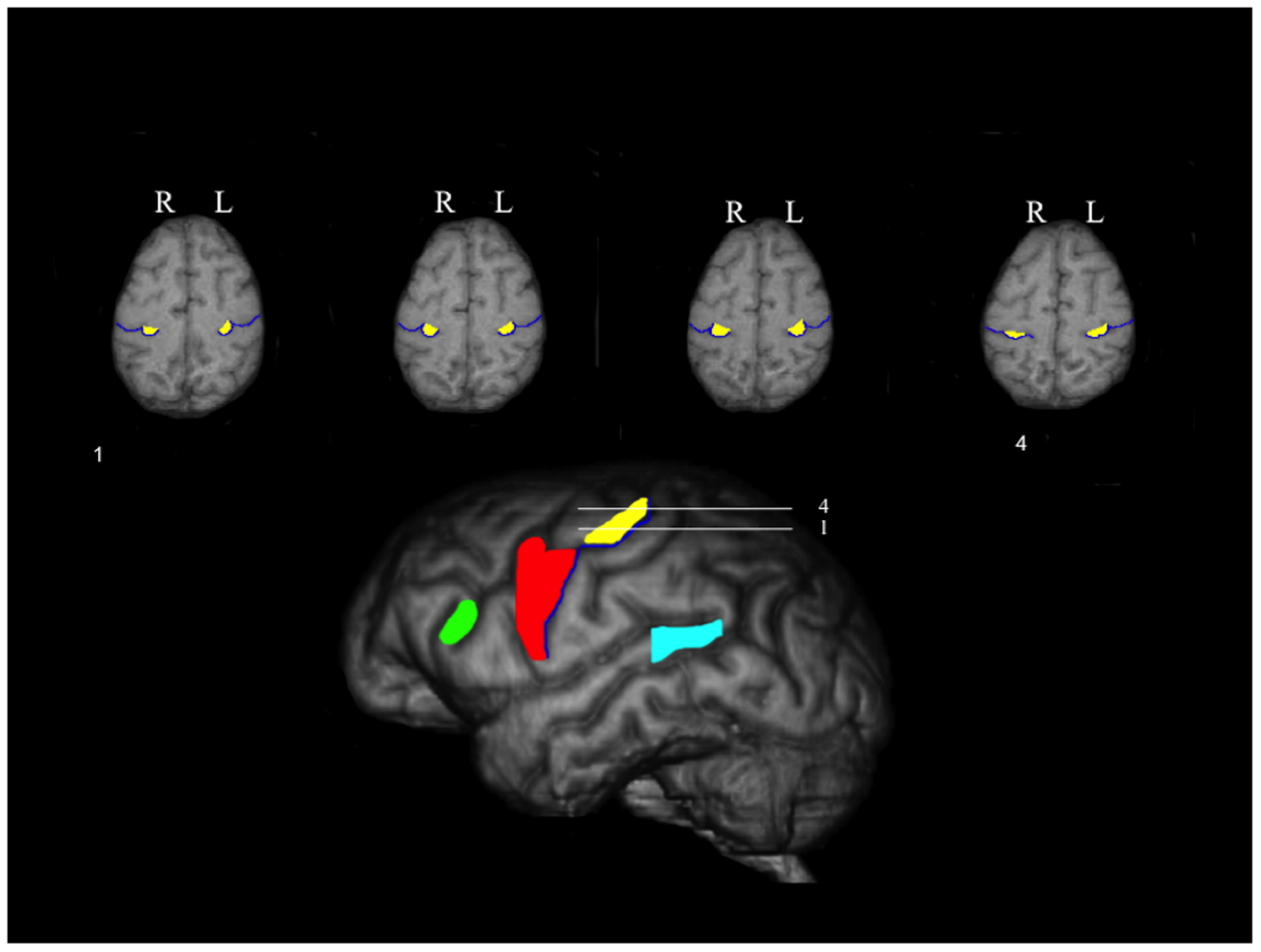
Axial views of 4 sequential images showing how the motor-hand area was traced in the individual brains. A 3D chimpanzee MRI scan is also shown to provide a reference point for where the motor-and is located on the dorsal-ventral plane.

In creating the probabilistic map of the KNOB, tracing from 62 chimpanzees were used including 21 males and 41 females. These 62 apes were selected because we could reliably trace the KNOB in both hemispheres whereas the KNOB was not clearly visible in one or both of the hemispheres in the remaining 8 apes. The KNOB was quantified separately for the left and right hemispheres, in the axial (transverse) plane on each spatially aligned brain (see Figure 4) following procedures previously used in human and chimpanzees brain specimens ([11], [24]. The dorsal and ventral edges of the knob served as markers for defining the boundaries of the area. The area of the entire knob was traced on each slice and hemisphere using a mouse-driven pointer (see Figure 4). The areas within the traced regions were subsequently summed to derive volumes of the KNOB for each hemisphere. These KNOB tracings for the right and left hemispheres were saved for each individual subject.

**Figure 5:**
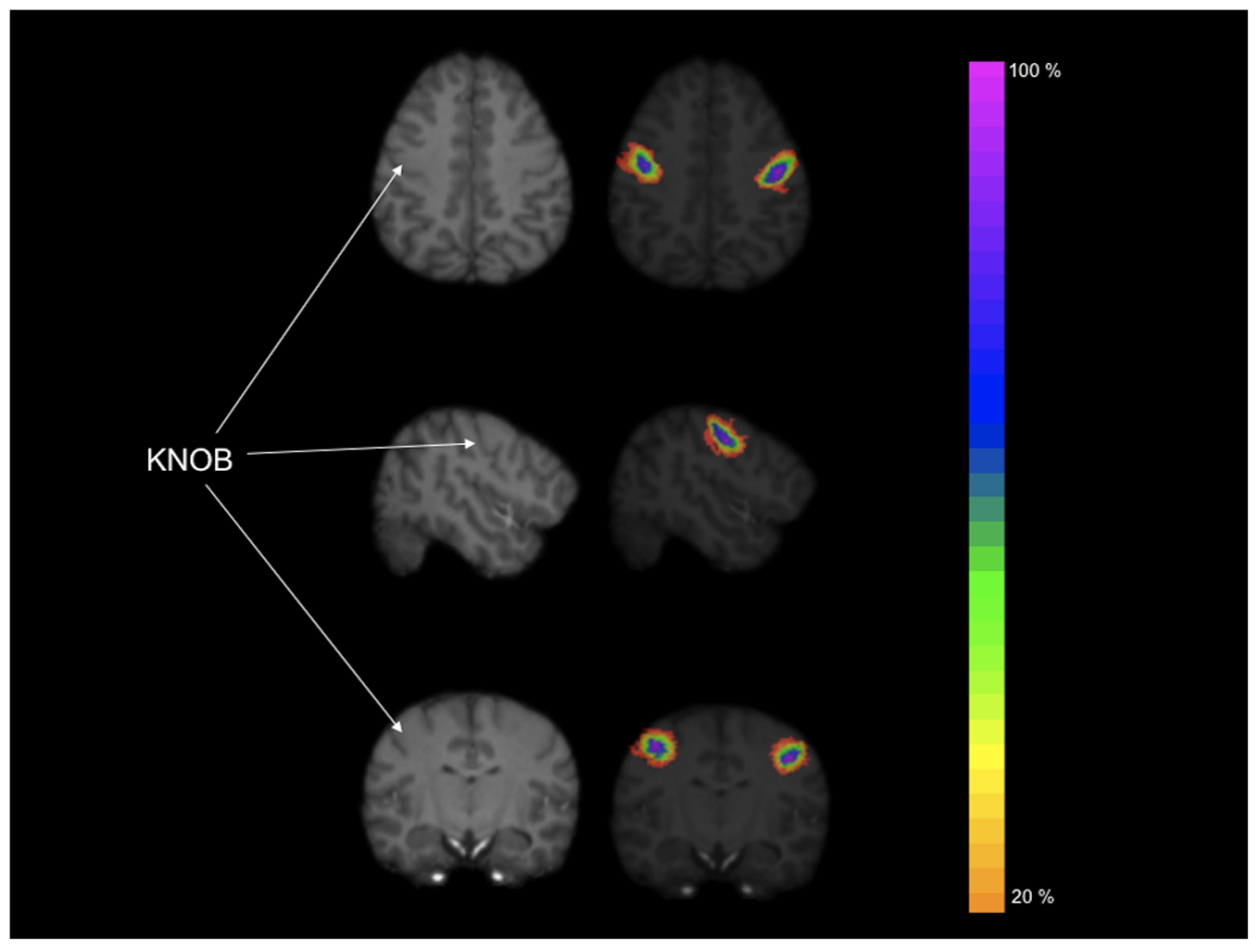
Axial, coronal and sagittal views of standard T1-weighted MRI scan and probabilistic map of the KNOB region. Different color intensities reflect the percentage of overlapping voxels across subjects.

The KNOB tracings were then summed across all 62 subjects and color-coded for consistency in the presence or absence of voxels (see Figure 5). The voxel values ranged from 1 to 62; a value of 62 represented voxels that were present in all subjects, whereas a value of 1 represented voxels which were present in only a single subject. The summed KNOB tracings were subsequently thresholded at three different criterion levels including 30%, 50% and 70% voxel overlap, respectively. The different thresholded maps represented the voxels within the individual KNOB tracings that were present in 30%, 50%, and 70% of the chimpanzee sample (i.e., common voxels in at least 19, 31 and 43 individuals, respectively). For each of the thresholded volumes, the resulting probabilistic map within the left and right hemisphere was outlined and saved for subsequent use with the segmented grey and white matter volumes.

### Grey and White Matter Segmentation and Quantification of the KNOB

For the grey matter (GM) and white matter (WM) analysis, each individual MRI scan was co-registered to the chimpanzee template. The MRI scans were then segmented into grey, white and CSF tissue using FSL (Analysis Group, FMRIB, Oxford, UK) [67], [68]. The KNOB volumetric maps for the left and right hemispheres that were derived for the 30%, 50% and 70% probabilistic maps were then applied to each of the co-registered GM and WM volumes. The volume of GM and WM found within the left and right hemispheres for the three different probabilistic maps were quantified across subjects.

### Performance Asymmetries in Grasping

Because the focus of the study was on the neural correlates of grasping, we also compared the KNOB asymmetries in relation to behavioral asymmetries in grasping performance when picking up small food items. We have previously reported that chimpanzees show population-level right hand biases in performance asymmetries when grasping small food items [35], [36]. Following the procedures used by Hopkins et al., [35], small food items were presented to the left and right hands of the chimpanzees and we recorded the number of errors they made when grasping the food items. The food items included small, quartered peanuts, mini m&m's, tiny-tarts, and small pretzel sticks. Each chimpanzee received 20 trials for the left and right hand for each food item with the exception of the quartered peanuts in which the apes received 40 trials for each hand. Thus, all the chimpanzees were required to grasp 60 food items with the left and right hands (total  = 120 trials) when the data were summed across the two studies. Based on these combined data, we computed a handedness index (HI) following the formula [HI  =  (#R - #L)/(#L + #R)] where #R and #L indicate the number of errors made by the left and right hand, respectively. For simplicity, we classified subjects as performing better with the left or right hand based on the sign of the HI value. Subjects with negative HI values were classified as performing better with the right hand while apes with positive HI values were judged to perform better with the left hand. Two chimpanzees performed equally well with both hands or could not complete testing and they were omitted from the analyses.

### Data analysis

For the probabilistic maps of the KNOB region, we calculated the percentage of GM and WM within the left and right hemisphere by calculating the GM and WM volume and dividing by the total volume of the probabilistic map for each threshold and hemisphere and multiplying by 100. We chose to use percentages of GM and WM rather than the raw volumes for each threshold because the size of the probabilistic object maps varied between hemispheres and we sought to adjust for those differences in estimating the amount of grey and white matter. All inferential statistics (ANOVA, t-tests) adopted an alpha of *p*<.05 (unless otherwise stated) and post-hoc tests, when necessary, were conducted using Tukey's Honestly Significant Difference (HSD) test.
